# Chronic Oral D-Galactose Induces Oxidative Stress but Not Overt Organ Dysfunction in Male Wistar Rats

**DOI:** 10.3390/cimb47030161

**Published:** 2025-02-27

**Authors:** Jelena Martinovic, Ivana Gusevac Stojanovic, Sladjan Nesic, Ana Todorovic, Katarina Bobic, Sanja Stankovic, Dunja Drakulic

**Affiliations:** 1 Department of Molecular Biology and Endocrinology, VINCA Institute of Nuclear Sciences—National Institute of the Republic of Serbia, University of Belgrade, 11000 Belgrade, Serbia; igusevac@vin.bg.ac.rs (I.G.S.); anato@vin.bg.ac.rs (A.T.); katarina.bobic@vinca.rs (K.B.); drakulic@vin.bg.ac.rs (D.D.); 2 Department of Pathology, Faculty of Veterinary Medicine, University of Belgrade, 11000 Belgrade, Serbia; sladjan@vet.bg.ac.rs; 3 Centre for Medical Biochemistry, University Clinical Centre of Serbia, 11000 Belgrade, Serbia; sanjast2013@gmail.com; 4 Faculty of Medical Sciences, University of Kragujevac, 34000 Kragujevac, Serbia

**Keywords:** oral d-galactose, aging, liver, heart, kidney, rat, oxidative stress

## Abstract

D-galactose (d-gal) plays numerous roles in the organism as an energy-providing nutrient and also an important constituent of the complex glycoconjugates. However, excessive amounts of d-gal activate alternative metabolic pathways that can lead to the development of a pro-oxidative environment. This feature is used in numerous aging studies which implied intraperitoneal (i.p.) or subcutaneous (s.c.) administration of d-gal for a prolonged time. The present study aims to investigate the systemic effects of orally administered d-gal (200 mg/kg and 500 mg/kg, dissolved in tap water, for 6 weeks) by analyzing oxidative stress parameters in the liver, kidney, and heart. For comparison with natural aging, the effects were studied in rats aged 12, 18, 24, and 30 months. In addition, histopathologic analyzes and serum biochemical measurements were performed to investigate the potential structural and functional organ damage induced by d-gal administration. Our findings show that chronic oral administration of d-gal induces oxidative stress in rat organs and mimics some aspects of natural aging similar to those of 30-month-old rats. Consistent with its primary role in galactose metabolism, the liver exhibited the most pronounced oxidative damage. However, despite the increased oxidative stress, only minor histopathological changes were observed, while organ function remained largely unaffected. Oral intake of d-gal was found to have milder effects compared to i.p. or s.c. injections, suggesting that this model may induce some features of natural aging but without overt organ dysfunction.

## 1. Introduction

D-galactose (d-gal) is an essential carbohydrate for cellular metabolism. It is mainly found in foods as a structural component of lactose and to a lesser extent in legumes and some fruits and vegetables. Galactose plays an important biological role beyond being a nutrient and metabolite, serving as a key structural component of glycoproteins and glycolipids [1]. As a monosaccharide, galactose is readily absorbed after digestion and enters the bloodstream, reaching target tissues where it is metabolized primarily through the Leloir pathway, which can lead to glycolysis to produce energy or participation in glycosylation [1,2]. However, with excessive amounts of galactose, alternative metabolic pathways that normally play only a minor role in cell biochemistry can be significantly boosted. This is the case with D-galactonate and galactitol biosynthesis, whereby the latter can act as a metabo-, neuro- and hepatotoxin [2]. Galactitol partially blocks the first step of the Leloir pathway, exacerbating galactose accumulation in the bloodstream. This also inhibits antioxidant enzymes and promotes free radical buildup, along with the osmotic changes that ultimately compromise the integrity of cell membranes, proteins, and mitochondrial DNA [2]. Galactitol has also been proposed as one of the links between galactose overexposure and cell aging. In animal studies, chronic intraperitoneal (i.p.) or subcutaneous (s.c.) treatment with d-gal resulted in oxidative damage to the brain and cognitive dysfunction, accompanied by several prominent features of the aging brain, such as impaired synaptic plasticity and neurogenesis. Chronic d-gal treatment has also been shown to be a reliable aging model to induce senescence in the liver [3], heart [4], and kidney [5,6,7,8].

The primary aim of this study was to determine if chronic treatment with d-gal dissolved in tap water induces age-related changes, as seen with other d-gal administration methods. The long duration of treatment (6–8 weeks) with i.p./s.c. d-gal is limited by cumulative irritation and needle damage, along with significant discomfort from animal restraint. Oral administration is considered more economical, convenient, and safe, though oral gavage poses risks like esophageal injuries, reflux, and aspiration pneumonia, which can affect experimental outcomes. While our previous study has documented the effects of oral d-gal intake on memory and redox balance without significant morphological changes in specific brain regions of rats [9], the present study aims to investigate the systemic effects by analyzing parameters of oxidative stress in vital organs—the liver, kidney, and heart. To identify similarities with natural aging processes, these effects were compared with those of rats at 12, 18, 24, and 30 months of age. In addition, histopathological examinations were performed and relevant organ function biochemical parameters in sera were measured to further elucidate the effects of the potential oxidative redox shift following d-gal administration.

## 2. Materials and Methods

### 2.1. Animal Treatment

All experimental procedures were conducted in accordance with the rules of the Ethical Committee for the Use of Laboratory Animals of the VINCA Institute of Nuclear Sciences—National institute of the Republic of Serbia, University of Belgrade (protocol number 02/11, license no J3 1/14) and the European Communities Council Directive (2010/63/EU, accessed on 31 January 2014).

Adult male Wistar rats (3 months old, 250–350 g) obtained from the local colony were kept under standard housing conditions: regular 12 h light/12 h dark cycle with constant temperature (21 ± 2 °C) and humidity while commercial rat pellet and tap water were available *ad libitum*. For the purpose of the 6-weeks-lasting experiment, rats were randomly assigned to three groups: control animals that drank pure tap water (Con, n = 8) and rats receiving either 200 mg/kg d-gal (200 mg/kg, n = 8) or 500 mg/kg d-gal (500 mg/kg, n = 8) dissolved daily in 120 mL of tap water. A detailed explanation and considerations regarding the dosage and route of administration are provided in the previous manuscript [9]. The in vivo observation involved daily food (g)/water (mL) intake, and physical health monitoring (consciousness, respiration, fur condition, skin, and piloerection (all scored as + or -), as well as weekly body weight (g)), while redox imbalance indicators along with structural and functional changes of the organs of interest (liver, heart, and kidney) were assessed ex vivo. In the first phase of the ex vivo study, the oxidative stress parameters in the liver, heart, and kidney (n = 5 animals/group) were examined. The second phase involved histopathological examination of the selected organs and a corresponding biochemical analysis of the serum (n = 3 animals/group). Additionally, naturally aged rats housed in standard housing conditions were sacrificed at ages 12, 18, 24, and 30 months, and parameters of oxidative stress in the liver, heart, and kidney (n = 5 animals/group) were measured.

Prior to sacrifice, blood glucose levels and urine parameters were determined using commercially available test strips (Accutrend glucometer, Roche Diagnostics GmbH, Rotkreuz, Switzerland and Insight Urinalysis Reagent Strips, Acon Laboratories, Inc., San Diego, CA, USA, respectively). The detection of bilirubin and urobilinogen (a breakdown product of bilirubin) in the urine is an early indication of liver disease. Urinary ketone and glucose is useful in managing and monitoring type 1 diabetes, while protein analysis is usually run to screen for kidney disease. Also, specific gravity evaluates the body’s water balance (hydration) and urine concentration and helps evaluate kidney functions and possible kidney diseases. The expected value ranges from 1.020 to 1.030.

### 2.2. Sample Preparations

The animals were decapitated with a Harvard Apparatus guillotine (Holliston, MA, USA) 24 h after treatments or upon reaching the appropriate age, and trunk blood was collected for serum preparation by centrifugation for 15 min at 3500 rpm (Heraeus Megafuge 2.0R, Hanau, Germany). The obtained sera were stored frozen (at −80 °C) until further analysis.

The liver, heart, and kidney were isolated and their weights were estimated for determining the organ’s fractional contribution, calculated as the ratio of wet tissue mass (mg) to body mass (g). For assessment of the oxidative stress parameters, 0.2 g of tissue was homogenized in cold phosphate-buffered saline (1:4 mass/volume) by an IKA T 10 Basic Ultra Turrax Homogenizer (IKA^®^-Werke GmbH & Co. KG, Staufen, Germany) and centrifuged for 45 min at 13,000× *g*, at 4 °C (Eppendorf microcentrifuge 5417R, Hamburg, Germany). The obtained supernatants were kept at −80 °C until processing.

The organs of interest from the remaining animals were placed in 10% formalin for histological analysis, while their sera were prepared for biochemical analysis of tissue function. Following the collection, the blood was centrifuged for 15 min at 3500 rpm (Heraeus Megafuge 2.0R, Germany), and the obtained sera were stored frozen (at −80 °C) until further analysis.

### 2.3. Measurement of Oxidative Stress Parameters

Pro-oxidant–antioxidant balance (PAB), the indicator of overall oxidative stress level that reflects the dynamic equilibrium between pro-oxidants and antioxidants was determined in the samples, as previously described by Guševac Stojanović and colleagues [10]. Briefly, upon the incubation and stopping of the reaction with HCl, the absorbance of the reaction mixture that contains tetramethylbenzidine (TMB) cation solution with TMB solution and sample or standard (hydrogen peroxide to uric acid in varying ratios) was measured at 450 nm using the microplate reader (Tecan Sunrise, Tecan Group Ltd., Männedorf, Switzerland). The values were reported in arbitrary units (HK), calculated as the percentage of hydrogen peroxide present in the standard solution.

Levels of advanced oxidation protein products (AOPPs), proteins that are modified through oxidative reactions predominantly by reactive oxygen species (ROS), were estimated in samples according to the protocol reported in detail earlier [10]. In short, the absorbance of the reaction mixture comprised of potassium iodide, phosphate buffer saline (pH 7.4), acetic acid, and sample or standard (various concentrations of chloramine T) was read at 340 nm. The AOPP concentration was expressed in µmol/L of chloramine T equivalents.

The extent of oxidative damage to cell membranes and lipids in the liver, heart, and kidney provoked by ROS was assessed using formerly established assays for MDA (Malondialdehyde) and HNE (Hydroxynonenal) [10]. The levels of these widely recognized markers of lipid peroxidation were evaluated by mixing the samples with working solution using hydrochloric acid to estimate the level of MDA and methane sulfonic acid and FeCl3 to determine HNE level. Following the incubation and centrifugation of the reaction mixture, the absorbance was measured at 580 nm, while the corresponding standard curves were used to quantify MDA and HNE concentrations in µM.

### 2.4. Histological Analysis

After the initial 72-h fixation in 10% formalin (pH 7), the tissue was further fixed in formalin for an additional 24 h to ensure complete stabilization. Following the fixation process, the tissue sections were embedded in paraffin and cut into 4 µm thick sections using a microtome. The thin sections were then mounted on glass slides and stained using the standard Hematoxylin and Eosin (HE) staining method that provides clear visualization of tissue architecture and cellular morphology under light microscopy. The cell nuclei are stained blue by Hematoxylin, while the cytoplasm and extracellular matrix are stained pink by Eosin.

### 2.5. Assessment of Sera Biochemical Parameters

The indicators of the health and performance of liver (aspartate transaminase (AST), alanine transaminase (ALT), alkaline phosphatase (ALP), and gamma-glutamyl transferase (GGT)), kidney (urea and creatinine), and heart (creatine kinase (CK) and troponin T (TnT)), as well as the general marker of tissue damage, lactate dehydrogenase (LDH), were routinely evaluated in serum by electrochemiluminescence immunoassays, using the RocheCobase601 automated analyzer (RocheDiagnostics GmbH, Penzberg, Germany). The levels of ALT, ALP, CK, GGT, and LDH are expressed in U/L, urea in mmol/L, and creatinine in µmol/L.

### 2.6. Data Analysis

All biochemical measurements were carried out in duplicate, and the mean recorded value was used. GraphPad Software v9 was used for all statistical analysis and graph creation. The oxidative stress parameters were analyzed using One-way ANOVA, followed by Tukey’s post hoc test. Biochemical and urine parameters were analyzed using the nonparametric Kruskal–Wallis test followed by Dunn’s multiple comparisons test. *p* < 0.05 was defined as statistically significant, and all data from the molecular studies were expressed as % of Con ± SEM.

## 3. Results

### 3.1. General Health Status

During the experiment, animals in the Con and both d-gal treated groups showed similar food/water consumption, and a gradual increase in body weight, with no statistical differences between groups. Moreover, other investigated indicators of physical health condition were comparable in all experimental groups. Among the urine parameters, the protein content was increased in the animals treated with d-gal, with statistical significance in the 500 mg/kg group compared to Con (H(2) = 5.664, *p* = 0.0492). This may be an early indicator of kidney function damage. Other urine parameters remained unchanged (Table 1). After a six-week treatment period there was no statistically significant difference in glycemic levels between animals that received d-gal treatments or water.

### 3.2. Parameters of Oxidative Stress

**Figure 1:**
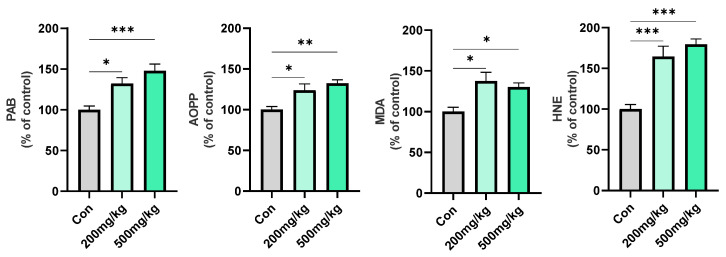
Parameters of oxidative stress (PAB, AOPP, MDA, HNE) in rat liver following oral d-gal treatments. Data are presented as the % of Con ± SEM. Symbols indicate significance from Con with *p* value thresholds of 0.05 (*), 0.01 (**), 0.001 (***).

In the liver, d-gal treatments affected PAB (F_2, 12_ = 12.48, *p* = 0.0012), AOPP (F_2, 12_ = 8.747, *p* = 0.0045), MDA (F_2, 12_ = 6.903, *p* = 0.0101), and HNE (F_2, 12_ = 22.09, *p* =< 0.0001). All the investigated parameters were significantly increased in the 200 mg/kg and 500 mg/kg groups compared to Con: PAB (0.0161, 0.0010, respectively), AOPP (0.0293, 0.0044, respectively), MDA (0.0112, 0.0382, respectively), and HNE (0.0007, 0.0001, respectively) (Figure 1.).

Aging also affected liver PAB (F_4, 20_ = 11.03, *p* < 0.0001), AOPP (F_4, 20_ = 5.458, *p* = 0.0039), MDA (F_4, 20_ = 17.29, *p* < 0.0001), and HNE (F_4, 20_ = 16.14, *p* =< 0.0001). In 30-month-old rats, all the investigated parameters were increased, while PAB, MDA, and HNE were significantly increased in 24-month-old rats, compared to Con and the remaining natural aging groups. In 18-month-old animals, a significant increase, compared to Con and 12-month-old rats, was observed only for HNE (Table 2).

**Figure 2:**
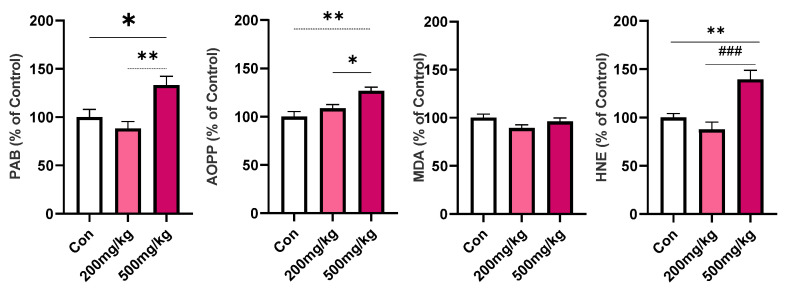
Parameters of oxidative stress (PAB, AOPP, MDA, HNE) in rat heart following d-gal treatments. Data are presented as the % of Con ± SEM, whereas the values of the Con group were set as 100%. Symbols indicate significance from Con with *p* value thresholds of 0.05 (*), 0.01 (**), and 200 mg/kg vs. 500 mg/kg with *p* value thresholds of 0.001 (^###^).

D-gal treatment affected PAB heart level (F_2, 12_ = 7.951, *p* = 0.0063), AOPP (F_2, 12_ = 9.223, *p* = 0.0037), and HNE (F_2, 12_ = 13.22, *p* = 0.0009). While 200 mg/kg dose did not provoke changes compared to Con, Post hoc Tukey’s indicate significantly higher PAB, AOPP, and HNE levels in 500 mg/kg compared to the 200 mg/kg group (*p* = 0.0061, 0.0360, 0.0009, respectively) and Con group (*p* = 0.0368, 0.0032, 0.0072, respectively) (Figure 2).

Natural aging, also, affected heart PAB (F_4, 20_ = 5.909, *p* = 0.0026), AOPP (F_4, 20_ = 7.798, *p* = 0.0006), and HNE (F_4, 20_ = 96.76, *p* =< 0.0001). Post hoc Tukey’s indicate significantly higher PAB and AOPP in 30-month-old rats compared to all other groups, while HNE was increased in 18- and 24-month-old rats as well (Table 3).

**Figure 3:**
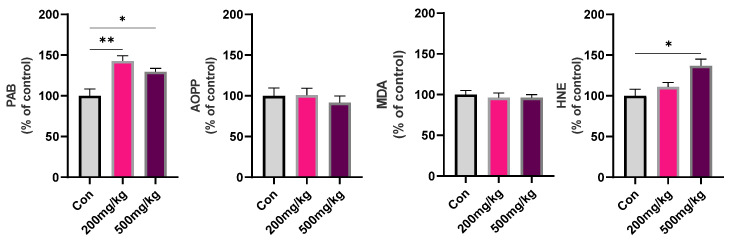
Parameters of oxidative stress (PAB, AOPP, MDA, HNE) in rat kidney following d-gal treatment. Data are presented as the % of Con ± SEM, whereas the values of the control group were set as 100%. Symbols indicate significance from Con with *p* value thresholds of 0.05 (*), 0.01 (**).

In the kidney, One-way ANOVA revealed a significant effect of the treatment on the PAB level (F_2, 12_ = 11.04, *p* = 0.0019) and HNE (F_2, 12_ = 6.657, *p* = 0.0113). Tukey’s test showed significantly increased PAB levels in 200 mg/kg and 500 mg/kg groups compared to Con (0.0017, 0.0202, respectively) and increased HNE levels in 500 mg/kg groups compared to Con (0.0103) (Figure 3).

Levels of PAB, AOPP, MDA, and HNE in the kidney were also affected by aging, (F_4, 20_ = 9.651, *p* = 0.0002), (F_4, 20_ = 6.603, *p* = 0.0015), (F_4, 20_ = 6.603, *p* = 0.0015), and (F_4, 20_ = 96.76, *p* < 0.0001), respectively. PAB was significantly increased in 24- vs. Con and 18-month-old rats (0.0372, 0.0062, respectively) and 30-month-old rats compared to Con, 12, and 18 months old (0.0018, 0.0409, 0.0003, respectively), while AOPP and MDA were significantly increased only in 24-month-old rats compared to other groups. HNE was increased from 18th month (Table 4.).

In summary, both 200 and 500 mg/kg d-gal doses increased oxidative stress parameters in the liver. In the heart and kidney, only the higher dose of d-gal led to a shift towards a pro-oxidative state. In the heart, both types of oxidative damage were observed, while, in the kidney, oxidative damage occurred in lipids but not in proteins. A significant increase in all oxidative stress parameters was observed exclusively in the tissues of the 30-month-old rats. In the 24-month-old rats, pro-oxidative conditions in the liver and kidney were associated with lipid oxidation but not protein damage. In the heart, changes in the redox balance were observed without concomitant lipid oxidation.

### 3.3. Histopathological Study and Biochemical Markers of Organ Function

During organ isolation, no macroscopic changes were observed. The relative weight of the investigated organs ((organ weight/body weight) × 1000) was similar in all investigated groups (Table 5).

**Figure 4:**
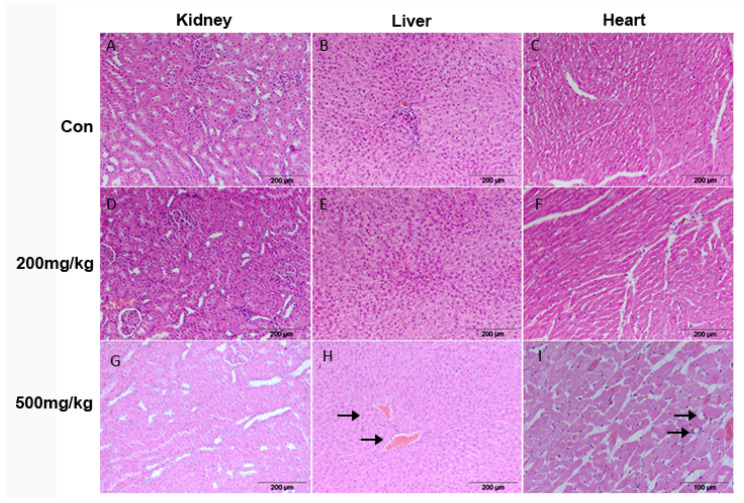
Representative micrographs of Hematoxylin and Eosin staining. Kidney—Con (**A**), 200 mg/kg (**D**), 500 mg/kg (**G**); Liver—Con (**B**), 200 mg/kg (**E**), 500 mg/kg ((**H**), black arrows, hyperemic blood vessels); Heart—Con (**C**), 200 mg/kg (**F**), 500 mg/kg ((**I**), black arrows, fat vacuoles).

Hyperemia is a non-specific finding in liver, heart, and kidney in all tissue sections of all groups of animals. The blood vessels in all tissues samples were dilated and filled with erythrocytes. In the kidneys of the experimental group treated with higher dose of d-gal, changes were observed in the tubulocytes, which have unclear borders, enlarged cytoplasm, and an almost imperceptible nucleus. The tubule lumen is narrowed. Individual dystrophic fibers are observed in the heart muscle. The fat vacuoles present as focal between muscle fibers in the heart (Figure 4).

**Figure 5:**
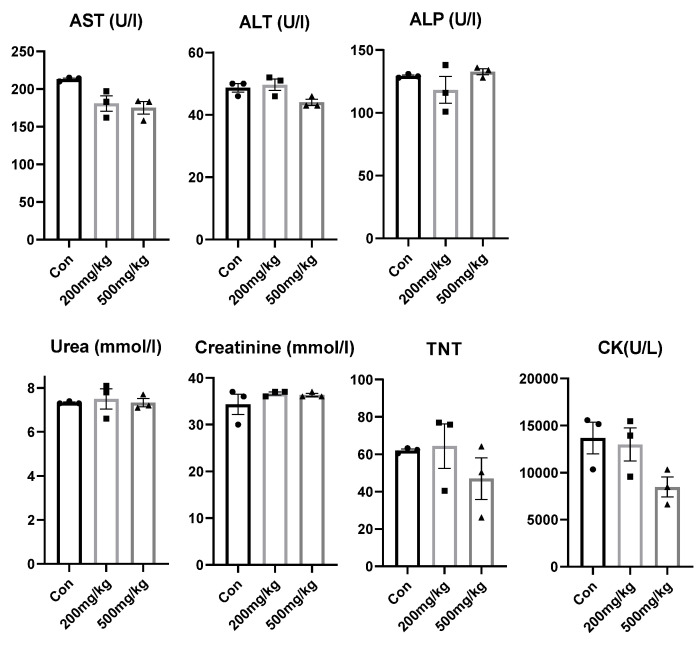
Serum biochemical markers of organ/tissue function following d-gal treatments. The diagrams show the distribution of the values of the individual animals. Abbreviation: aspartate aminotransferase (AST), alanine aminotransferase (ALT), alkaline phosphatase (ALP), creatine kinase (CK), troponin T (TnT).

Simultaneous analysis of biochemical markers in the serum of animals whose organs were subjected to histopathological analysis revealed a statistically significant effect of d-gal treatment on AST levels (H(2) = 5.535, *p* = 0.05). However, the Dunn’s multiple comparisons test showed no significance between the groups. Although a decreasing trend of LDH and CK was observed in the 500 mg/kg group, the statistics showed no significance (Figure 5). GGT remained under 4 in all experimental groups.

## 4. Discussion

In numerous studies, authors have reported the alternations of components of the antioxidant defense system and generation of free radicals but also the oxidative damage of macromolecules following s.c. and i.p. d-gal treatment. In the liver, heart, and kidney of rats, increased levels of ROS, MDA and nitric oxide as well as a decrease in superoxide dismutase, catalase, glutathione, and total antioxidant capacity were found [3,4,7,8,11,12,13]. Our current study reports, for the first time, that oral d-gal treatments also shift the redox balance in these organs towards a pro-oxidant state, but not to the same extent. In the liver, a pro-oxidative state was achieved at both 200 and 500 mg/kg d-gal, as indicated by elevated parameters for oxidative damage to lipids and proteins. As literature data point to the liver as the predominant organ of accumulation of ingested galactose following transport through the portal vein after consumption [2], the most intense oxidative response in the liver is expected. The remaining organs were not affected to the same extent—in the heart and kidney, only the higher dose of d-gal triggered a shift toward a pro-oxidant state. While, in the heart, both types of oxidative damage were observed, in the kidney, however, oxidative damage to lipids and not to proteins was observed. It was significant to further investigate whether the same pattern was evident in naturally aging rats, as the oxidative stress and inflammation are key mechanisms that significantly contribute to the progression of organ diseases in aging [14]. Interestingly, our results from naturally aged animals suggest that macromolecules are not as sensitive to oxidative damage as one might expect. A significant increase in all oxidative stress parameters examined was observed exclusively in the tissues of 30-month-old rats. In the 24-month-old group, an organ-specific response to aging was detected—pro-oxidant conditions in the liver and kidney were associated with lipid oxidation but not with protein damage. In the heart, changes in redox balance were observed without concomitant lipid oxidation. This suggests that, although aging rats are predisposed to oxidative stress, the biological response is variable and organ-specific. It is worth noting that in all three organs of rats in older and late adulthood, the same pattern was observed: unchanged MDA levels but increased levels of HNE. This finding is particularly noteworthy because MDA is a well-known byproduct of lipid peroxidation, while HNE has been shown to also have significant biological activity. Unlike MDA, HNE can act as a signaling molecule, influencing stress response pathways and the activation of transcription factors [15,16]. Therefore, the increase in HNE levels, even without a corresponding rise in MDA, may not reflect oxidative damage per se but could indicate an adaptive or regulatory response that warrants further investigation [17].

Oxidative stress is recognized as a crucial factor in the development of various diseases, but generalizing the significance of specific biomarkers of oxidative stress can be challenging due to their context-dependent nature [18]. Our study emphasizes the importance of assessments at both molecular and cellular levels and observation of microscopic changes to comprehensively understand the effects of d-gal treatment and aging on organs function and structure. We observed increased levels of oxidative stress parameters; however, the statistically significant effect of d-gal treatment was only seen in AST levels, while other serum biochemical parameters remained statistically unchanged. Some studies have reported unchanged or increased levels of liver enzymes, such as AST and ALT, as a result of s.c. d-gal treatment [19,20,21]. Our study showed a decreasing trend in AST and ALT levels in the d-gal groups. These changes can be observed in chronic kidney disease with/without end-stage renal disease [22,23], and, although rare, low AST may signal vitamin B6 deficiency [24] that is more likely to occur in the elderly and people with underlying health conditions such as liver, kidney, or inflammatory diseases. Combined with detected proteinuria, these changes suggest potential alterations in kidney function; however, other serum kidney parameters such as urea and creatinine remained unchanged, indicating the need for further analyses for a definitive conclusion. Could proteinuria be an early marker of kidney dysfunction that has yet to show up in the traditional kidney function markers?

In the d-gal induced aging model, i.p. or s.c. administration in rats resulted in profound structural damage to hepatocytes, including apoptosis, degeneration, and necrosis, as well as in increase in markers of liver function such as AST, ALP, and GGT [3]. Renal injury has also been confirmed by increased serum creatinine and urea levels, with histopathological observations of tubular damage, necrotic epithelial cells, and inflammatory infiltrates [6,7]. Cardiac aging models induced by d-gal have demonstrated cardiac hypertrophy, increased inflammatory cells, and adipose tissue hyperplasia [4]. Unlike i.p. and s.c. treatment, oral treatment used in our study is associated with unspecific histological changes. However, together with unchanged markers of organ function, this indicates no effect of oral d-gal treatment on structure and function of the investigated organs. Although oxidative stress markers indicated a shift toward a pro-oxidant state in certain organs, the unchanged serum markers of organ function and absence of significant histological changes suggest that oral d-gal treatment may not lead to significant functional or structural damage at the doses and time points studied. These findings underscore the need to further investigate the long-term impacts of oral d-gal treatment and its potential role in age-related oxidative stress, emphasizing the need for both biochemical and histological evaluations to capture a comprehensive picture of organ health.

## 5. Conclusions

Our study demonstrates that the route and dosage of d-gal are critical factors influencing its effects on redox balance and oxidative stress, which are key components of the aging process. Chronic oral administration of d-gal induces oxidative stress in investigated rat organs, thus mimicking certain aspects of natural aging observed in 30-month-old rats. Consistent with its primary role in galactose metabolism, the liver exhibited the most pronounced oxidative damage. Notably, despite the increased oxidative stress, only minor histopathological changes were detected, while organ function remained largely unaffected.

The importance of our results extends beyond the scope of nutrition. By using oral d-gal treatment as a tool to induce a pro-oxidant state in rats without causing overt organ dysfunction, we provide a potential model for studying the early stages of oxidative stress-related aging that is less invasive than s.c. and i.p. This model may be valuable for testing interventions aimed at mitigating oxidative damage and prolonging organ health. In summary, our work not only contributes to the existing literature on the effects of d-gal consumption but also provides a novel perspective on its potential role in aging research, highlighting the complex interplay between diet, redox balance, and organ structure and function.

## 6. Experimental Consideration

We would like to emphasize that the primary aim of this study was to determine whether chronic treatment with d-gal dissolved in tap water can induce pro-oxidant changes similar to those observed in naturally aged rats or in studies involving other ways of d-gal application. Once we noticed pro-oxidant changes in d-gal treated animals, our further aim was to determine whether this alteration leads to morphological and functional changes in the observed organs.

To avoid any overstatement, we would like to point out the limitations of this study —the number of animals in the second part of the ex vivo study (histopathological and biochemical serum analysis). Although the histopathological analysis showed no differences between the animals of the same group, the serum analysis of some parameters showed a dispersity in values within the group. Therefore, a larger number of animals per group is required to draw definite conclusions about the biochemical changes in serum that occur as a result of oral d-gal treatment.

## Figures and Tables

**Table 1:** Urine parameters and serum glucose level following oral d-gal treatment. Data are presented as the mean ± SEM. Symbol indicates significance from Con with *p* value thresholds of 0.05 (*).

	Con	200 mg/kg	500 mg/kg
Ketones (mmol/L)	12.5 ± 2.5	23.3 ± 8.3	15.0 ± 0.0
Urobilinogen (µmol/L)	0.2 ± 0.0	0.2 ± 0.0	0.2 ± 0.0
Proteins (g/L)	47.5 ± 17.5	100 ± 0	166.7 ± 66.6 *
Specific gravity	1.02	1.02	1.02
Bilirubin (µmol/L)	negative	negative	negative
pH	7.3	7.5	8
Serum Glucose (mmol/L)	6.9 ± 0.2	6.6 ± 0.2	6.3 ± 0.1

**Table 2:** Parameters of oxidative stress (PAB, AOPP, MDA, HNE) in the liver of naturally aged rats. Data are presented as the % of Con ± SEM, whereas the values of the Con group were set as 100%. Symbols indicate significance from Con (*), 12 months old (#), and 18 months old ($). *p* value thresholds of 0.05, 0.01, 0.001, and 0.0001.

	PAB	AOPP	MDA	HNE
Con	100 ± 4.8	100 ± 3.9	100 ± 5.4	100 ± 5.6
12 months old	80.5 ± 6.8	113.5 ± 4.8	100.6 ± 2.9	104.4 ± 12.3
18 months old	91.16 ± 7.4	122.3 ± 6.5	115.1 ± 3.3	160.3 ± 13.9 * ^#^
24 months old	132.3 ± 8.0 * ^### $$^	115.8 ± 9.9	129.7 ± 2.1 *** ^###^	201.0 ± 6.2 **** ^####^
30 months old	134.4 ± 9.1 * ^### $$^	140.9 ± 5.0 ** ^#^	135.3 ± 4.9 **** ^#### $^	188.4 ± 16.3 *** ^###^

**Table 3:** Parameters of oxidative stress in heart of naturally aged rats. Data are presented as the % of Con ± SEM, whereas the values of the control group were set as 100%. Symbols indicate significance from Con (*), 12 months old (^#^), 18 months old ($), and 24 months old (%) rats. *p* value thresholds of 0.05, 0.01, 0.001, and 0.0001.

	PAB	AOPP	MDA	HNE
Con	100 ± 8.2	100 ± 5.5	100 ± 3.7	100 ± 8.1
12 months old	115.8 ± 2.6	91.5 ± 4.9	104.7 ± 6.8	125.6 ± 10.7
18 months old	114.3 ± 7.3	96.4 ± 5.8	111.5 ± 6.8	174.2 ± 13.0 *** ^#^
24 months old	116.8 ± 8.2	86.4 ± 7.4	118.4 ± 7.6	191.8 ± 14.0 **** ^##^
30 months old	148.3 ± 8.5 ** ^# $ %^	129.6 ± 6.4 * ^## $ %^	103.4 ± 6.1	368.9 ± 5.5 **** ^#### $$$$ %%%%^

**Table 4:** Parameters of oxidative stress in the kidney of naturally aged rats. Data are presented as the % of Con ± SEM, whereas the values of the control group were set as 100%. Symbols indicate significance from Con (*), 12 months old (#), and 18 months old ($) rats. *p* value thresholds of 0.05, 0.01, 0.001, and 0.0001.

	PAB	AOPP	MDA	HNE
Con	100 ± 8.4	100 ± 3.9	100 ± 9.5	100 ± 8.1
12 months old	113.9 ± 6.8	113.5 ± 4.8	115.5 ± 10.1	125.6 ± 10.7
18 months old	91.9 ± 4.1	122.3 ± 6.5	126.7 ± 9.8	174.2 ± 13.0^** #^
24 months old	130.5 ± 5.1 * ^$$^	115.8 ± 9.9	169.4 ± 10.6 *** ^# $^	191.8 ± 14.0 **** ^## $$$$^
30 months old	144.0 ± 8.8 ** ^# $$$^	140.9 ± 5.0 ** ^#^	132.0 ± 10.3	368.9 ± 5.5 **** ^#### $$$$^

**Table 5:** Body weight (g) and relative organ weight ((organ weight/body weight) × 1000, g) in experimental groups.

	Body Weight	Liver	Heart	Kidney
**Con**	435.0 ± 5.0	32.5 ± 2.3	2.8 ± 0.0	7.1 ± 0.1
**200 mg/kg**	433.5 ± 12.5	31.9 ± 0.7	3.0 ± 0.1	6.4 ± 0.2
**500 mg/kg**	400.0 ± 0.0	29.4 ± 0.5	2.97 ± 0.1	6.4 ± 0.0

## Data Availability

The data are contained within the article.
